# Two Months of War Work at Manchester: The Royal Infirmary's Aid for the Wounded

**Published:** 1914-12-19

**Authors:** 


### TWO MONTHS OF WAR WORK AT MANCHESTER.

### The Royal Infirmary's Aid for the Wounded.

On the outbreak of the war the Board of Management of the Manchester Royal Infirmary offered to the War Office fifty beds for the treatment of seriously wounded or ailing soldiers, and at the same time intimated that they were prepared to increase this offer to approximately 200 beds if the necessity arose. The War Office accepted, and the Board of Management proceeded to equip the fifty beds in the fifth medical unit, which was provided in excess of the accommodation immediately needed when the building was erected in 1908.

### The Earliest Arrivals.

The first reception of wounded was on October 18 and consisted of fifty Belgian soldiers transferred from the 2nd Western General Hospital. They were an exceedingly nice lot of men, and it was astonishing how soon they made themselves at home. Fortunately two of their number spoke English very well, and there were a few linguists amongst the resident staff and nurses, and one or two French and Flemish speaking friends from the city also assisted, and it was not long before all were fed, registered, and settled for the night.

Very soon an intimation was received from the military authorities that additional accommodation would be acceptable, and a further forty-eight beds were at once arranged for in the infirmary and occupied by British soldiers, followed a fortnight later by the opening of the new central branch, which had just been completed in the centre of the city as a receiving house for accidents and for the treatment of out-patients. This building consists of a complete hospital with small ward accommodation for fourteen patients, residential quarters for the necessary surgical, nursing, and domestic staffs, an out-patient hall capable of seating 400 patients, with consulting rooms adjacent, an accident department with examination cubicles, an operation theatre, *x*-ray room, etc. It was nearing completion on the outbreak of the war, and in offering to extend to 200 beds the Board of Management had in contemplation the conversion of the out-patient hall and consulting rooms of this building into wards, and when the military authorities asked for additional accommodation, the extra equipment was speedily obtained and the new branch opened by the reception of sixty British soldiers on November 6. The out-patient hall and consulting rooms make useful wards, and this branch institution of the Manchester Royal Infirmary is one of which Mancunians will be proud. Following upon the occupation of the central branch fifty additional beds were provided in large day-rooms, etc., attached to the surgical units in the infirmary itself, making 208 beds devoted to military patients, and 736 beds altogether in occupation.

It will be fully realised that this great increase in the accommodation meant large additions to the staff of all ranks, and coming as it did after no fewer than sixteen of the honorary, resident, and non-resident medical staffs had been called to the Front, and many nurses had been mobilised for Territorial service at the 2nd Western General Hospital, and many others sent to augment Queen Alexandra's Imperial Nursing Service, it was no light task to fill the vacancies. This was, however, successfully accomplished, and the patients are not at all backward in expressing their appreciation of the care and attention they are receiving and the comfort of their surroundings, while the staff of the infirmary of all ranks are deeply impressed by the cheery way in which the British soldiers bear their sufferings and make light of the dangers through which they have passed.

Since October 18, when the first fifty beds were opened, 502 military patients have been received, the great majority of whom were admitted direct from the hospital trains. Midnight or 2 or 5 A.M. are the favourite times for trains to arrive, and the prompt removal of the wounded from the trains to the various hospitals in the town by the fleet of Red Cross motor ambulances, Police motor and horse ambulances, the infirmary motor ambulance, and a large number of private motor-cars is one of the features of the work—150 to 200 wounded, including a large proportion of "stretcher" cases, being safely housed in les than an hour.

### Administration Problems.

Immediately on reception the wounded are fed, and it is astonishing how ready they are for the hot tea and huge piles of bread, butter, and jam which are provided in readiness, then follows a good cleaning up and attention to dressings; and, as many of the patients have not had their clothing off for weeks, it is easily understood how acceptable are the cleaning up, clean linen, and comfortable beds. Registration and diagnosis immediately follow to enable the nominal roll to be promptly sent to the military authorities.

All clothing has then to be listed, disinfected, and surveyed, the unserviceable being placed on one side for condemnation and destruction, requisitions to replace deficiencies prepared and forwarded to the military authorities, pay-books collected, and the numerous details necessary to comply with military regulations carried out.

### Patients and Theatre Work.

The *x*-rays play an important part in the treatment of the wounded. Bullets, pieces of shrapnel and shell need locating carefully before the operation for removal is attempted, and many exceedingly interesting radiographs are taken. The surgeons, too, have a busy time, and the nine operation theatres are kept fully occupied during the forenoon (the honorary medical and surgical staffs' attendance at the hospital being in the morning, except, of course, for emergency work). The patients are very keen to take possession of the pieces of metal which the surgeons extract, so as to show their friends the cause of their injuries. One unfortunate was simply riddled with bullets, having no fewer than sixty-four wounds, in spite of which he has done well.

The treatment of upwards of 200 wounded in addition to the ordinary work of the hospital keeps the entire staff more than busy, but the work is full of interest, and it is a comfort to feel that, although it is impossible for the whole staff to go to the Front, yet those who are remaining at home are able to share in some measure in the work of the great war machine, and are able to perform an immense service to the State.

